# From the Desk of Managing Editor

**DOI:** 10.4103/0971-6580.75842

**Published:** 2011

**Authors:** A. B. Pant

**Affiliations:** Scientist, Indian Institute of Toxicology Research (IITR), Lucknow, India. E-mail: abpant@rediffmail.com

Dear Friends,

At the outset I extend my candid wishes to you and your family members for an eventful and prosperous New Year. Beginning January 1, 2011, I have taken over the responsibility of becoming the ‘Managing Editor’ for ‘Toxicology International,’ the official journal of the Society of Toxicology, India. I take this opportunity to express my gratitude to my predecessors for their vision and untiring efforts, which have given a strong base for setting the high standards that the journal continues to uphold, with significant visibility among scientists, academia, practitioners, and research students. I understand the challenges to compete with established journals in the area, in the present scenario, however; collective efforts of membership will help us reach the heights.

Being a multidisciplinary forum for toxicology research, we welcome for publication, original research articles, with sufficient importance, novelty, and breadth of interest. In addition to that, articles presenting hypotheses and commentaries addressing current issues, which are of immediate interest to other investigators, are also welcome. Updates pertaining to areas of current importance and interests are also published in the form of mini-reviews. Besides clinical reports, occupational and safety evaluation, legal, risk, and hazard assessment, and impact of toxicants on humans, animals, and environment of sufficient novelty to warrant rapid publication, are also considered.

My goal is to continue with the policies developed by the earlier Editors, while striving to raise the standards, reputation, and visibility even further. In order to achieve this, the Advisory Board has been expanded, by including seasoned and active scholars working in diversified areas of toxicology. This will facilitate improvement in review processing for each submitted manuscript, by including one member, and eventually it would help in enhancing the consistency of better quality in the articles. I wish to develop a tradition of publishing special issues with a specific focal theme of high priority and relevance falling within the scope of the journal, which will further increase the journal’s strength, visibility, and circulation. A new feature, to provide a forum for the discussion and interpretation of data published in earlier issues of the journal, is also under consideration. We have achieved success in a difficult, but important task, of reducing the time between submission and publication through online electronic manuscript submission and a peer review system (http://www.journalonweb.com/ti/), which allows submission of articles with tracking of its progress till proof. It will be our endeavor to increase the number of issues in future. As you are aware that the journal is indexed at various sites, including ‘Pubmed’, it is high time to brain storm to have to procure an impact factor to improve the ranking of our journal among the journals published in the area of Toxicology.

I publically express my sincere thanks to all the members of the Editorial and Advisory Board, both past and present, the reviewers, authors, and all the dedicated personnel at Medknow Publications, in advance, for their contribution, cooperation, and services. Perhaps you may find some of my goals to be ambitious and far reaching, but I am confident that with your help and cooperation I will be able to achieve these targets.

I look forward to continuing and building on the outstanding work of my predecessor, and the many other visionary and skilful people involved in creating and enriching this journal and guiding its development since the day of its inception. It is an appeal to forward suggestions to improve the quality of the journal, to deliver the best to the scientists and the society.

Best wishes,

